# Oligodendrocyte-derived IL-33 regulates self-reactive CD8^+^ T cells in CNS autoimmunity

**DOI:** 10.1084/jem.20241188

**Published:** 2025-04-14

**Authors:** Nicolas Fonta, Nicolas Page, Bogna Klimek, Margot Piccinno, Giovanni Di Liberto, Sylvain Lemeille, Mario Kreutzfeldt, Anna Lena Kastner, Yusuf I. Ertuna, Ilena Vincenti, Ingrid Wagner, Daniel D. Pinschewer, Doron Merkler

**Affiliations:** 1 Department of Pathology and Immunology, University of Geneva, Geneva, Switzerland; 2 Division of Clinical Pathology, Geneva University Hospital, Geneva, Switzerland; 3 Division of Experimental Virology, Department of Biomedicine, University of Basel, Basel, Switzerland

## Abstract

In chronic inflammatory disorders of the central nervous system (CNS), tissue-resident self-reactive T cells perpetuate disease. The specific tissue factors governing the persistence and continuous differentiation of these cells remain undefined but could represent attractive therapeutic targets. In a model of chronic CNS autoimmunity, we find that oligodendrocyte-derived IL-33, an alarmin, is key for locally regulating the pathogenicity of self-reactive CD8^+^ T cells. The selective ablation of IL-33 from neo–self-antigen–expressing oligodendrocytes mitigates CNS disease. In this context, fewer self-reactive CD8^+^ T cells persist in the inflamed CNS, and the remaining cells are impaired in generating TCF-1^low^ effector cells. Importantly, interventional IL-33 blockade by locally administered somatic gene therapy reduces T cell infiltrates and improves the disease course. Our study identifies oligodendrocyte-derived IL-33 as a druggable tissue factor regulating the differentiation and survival of self-reactive CD8^+^ T cells in the inflamed CNS. This finding introduces tissue factors as a novel category of immune targets for treating chronic CNS autoimmune diseases.

## Teaser

Oligodendrocyte-derived IL-33 regulates self-reactive CD8^+^ T cells in CNS autoimmunity. IL-33 ablation reduces persistence and TCF-1^low^ effector T cell generation, mitigating disease. Local IL-33 blockade via delivery of a decoy receptor improves outcomes, highlighting tissue factors as druggable targets for chronic CNS autoimmune disorders.

## Introduction

CD8^+^ T cells mediate pathogen elimination and tumor immunosurveillance; however, when aberrantly activated, they can contribute to the pathogenesis of a wide spectrum of chronic autoimmune diseases (Collier et al., 2021). In multiple sclerosis (MS), a chronic autoimmune disease of the central nervous system (CNS), CD8^+^ T cells constitute the majority of lymphocytes found in active demyelinating CNS lesions, and they are considered critical effectors of tissue damage (Friese and Fugger, 2005; Mockus et al., 2021). Both circulating CD8^+^ effector memory cells and tissue-resident memory T cells (T_RM_) are important for perpetuating inflammation within the affected organ (Clark, 2015). While effector memory cells are in equilibrium with the circulation, T_RM_ cause tissue damage and sustain localized inflammation in a compartmentalized manner (Frieser et al., 2022; Vincenti et al., 2022).

Under conditions of prolonged antigen exposure, such as in chronic viral infections and cancer, tissue-infiltrating CD8^+^ T cells undergo distinct differentiation reprogramming, which may lead to altered T cell effector function, often referred to as exhaustion (McLane et al., 2019). While mitigating excessive CD8^+^ T cell activity and tissue damage, this adaptation can further contribute to the persistence of autoimmune responses within the affected organ (Grebinoski et al., 2022). Among various factors for the persistence and the potential to sustain inflammation of autoreactive CD8^+^ T cells, the T cell–specific transcription factor (TF) T cell factor 1 (TCF-1, encoded by *Tcf7*) has garnered attention (Gearty et al., 2022). In acute viral infection, CD8^+^ T cell–intrinsic TCF-1 is crucial for the development and preservation of functional memory CD8^+^ T cells (Zhou et al., 2010). Likewise, TCF-1^high^ CD8^+^ T cells retain stem-like properties when chronically stimulated, demonstrated by their sustained proliferative potential, self-renewing capacity, and the ability to differentiate into terminal effector cells (Utzschneider et al., 2016; Wu et al., 2016). Extrinsic factors such as IL-12 strongly regulates the stemness function of CD8^+^ T cells (Danilo et al., 2018), yet the local cues within the target tissue governing the longevity and the destructive potential of autoreactive CD8^+^ T cells are not well understood.

IL-33, an IL-1 family member, acts as a damage-associated molecular pattern or “alarmin”, regulating a broad range of inflammatory processes (Liew et al., 2016). It is released upon tissue damage in the context of various pathological conditions, such as viral infections (Kallert et al., 2017; Bonilla et al., 2012), asthma (Préfontaine et al., 2009), allergies (Chan et al., 2019), and sepsis (Alves-Filho et al., 2010). The cytokine is sensed by immune cells (Cayrol and Girard, 2014) expressing the IL-33 receptor ST2 (IL-1RL1), among them Th1- and Th2-differentiated CD4^+^ (Baumann et al., 2015; Löhning et al., 1998) and CD8^+^ T cells (Bonilla et al., 2012).

In CD8^+^ T cells, IL-33 enhances the antiviral functionality of effector CD8^+^ T cells during acute and latent infection as well as upon vaccination (McLaren et al., 2019; Kallert et al., 2017; Villarreal et al., 2014; Bonilla et al., 2012). Moreover, it preserves and promotes the stemness of memory-like CD8^+^ T cells in systemic viremic infection (Marx et al., 2023). In the CNS, IL-33 serves immunomodulatory functions, where it is expressed at high levels (Fairlie-Clarke et al., 2018). The primary sources of IL-33 in the CNS are astrocytes during development (Vainchtein et al., 2018) and oligodendrocytes in adulthood (Gadani et al., 2015).

IL-33/ST2 signaling promotes microglial phagocytosis, which is essential for proper synapse elimination and formation during brain development (Xiong et al., 2022). Furthermore, astrocyte-derived IL-33 induces interactions between microglial processes and dendritic spines, enhancing synaptic engagement and plasticity during early thalamogenesis (Xiong et al., 2022) (Vainchtein et al., 2018). Neurons have been described not to express IL-33 (Gadani et al., 2015; Rao et al., 2022; Fairlie-Clarke et al., 2018), while one study showed that neurons in the hippocampus can express IL-33 (Nguyen et al., 2020).

In chronic neuroinflammatory CNS diseases like MS, IL-33 is increased in both the periphery and the CNS compared with healthy controls (Jafarzadeh et al., 2016; Allan et al., 2016). IL-33’s role in this disease remains, however, unclear, showing both pro-inflammatory and anti-inflammatory effects (De la Fuente et al., 2015; Liew et al., 2016), and the role of glial cell-derived IL-33 on autoreactive CNS-infiltrating CD8^+^ T cells has remained unexplored.

Exploiting a CD8^+^ T cell–driven experimental autoimmune mouse model for chronic CNS inflammation, we studied how oligodendrocyte-derived IL-33 affects the behavior and differentiation of CNS-infiltrating self-reactive CD8^+^ T cells and their resulting tissue damage. Our study reveals a role of IL-33 as an oligodendrocyte-derived tissue factor sustaining the pathogenic responses of autoreactive CD8^+^ T cells.

## Results and discussion

### Oligodendrocytes-derived IL-33 enhances tissue damage in CNS autoimmune disease

**Figure 1.**
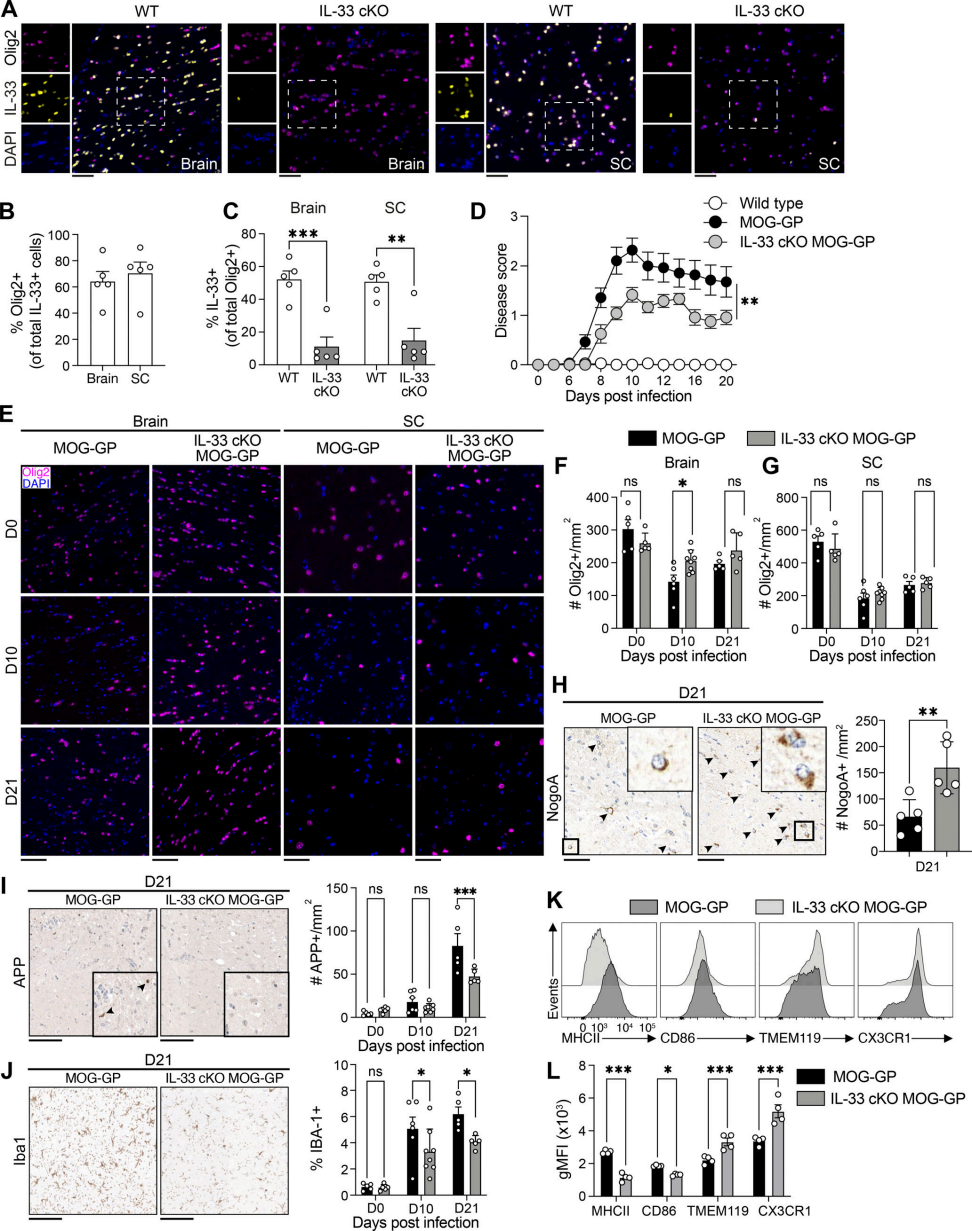
**Oligodendrocytes-derived IL-33 enhances tissue damage in CNS autoimmune disease. (A)** Brain and spinal cord (SC) sections of WT and IL-33 cKO mice stained for Olig2, IL-33, and Dapi. Scale bars, 50 μm. **(B)** Percentage of Olig2^+^ cells among total IL-33^+^ cells in WT brain and SC tissues. **(C)** Percentage of IL-33^+^ cells among total Olig2^+^ cells in WT and IL-33 cKO brain and SC tissues. **(D–G)** WT, MOG-GP, and IL-33 cKO MOG-GP mice were infected i.v. with 10^4^ PFU rLCMV-GP33. **(D)** EAE disease course (*n* = 10 WT mice; *n* = 14 MOG-GP mice; *n* = 12 IL-33 cKO MOG-GP mice); clinical scores are expressed as mean ± SEM. **(E)** Representative immunostainings for Olig2 and Dapi on brain (left) and SC (right) sections of MOG-GP and IL-33 cKO MOG-GP mice at indicated time points after infection. Scale bars, 50 μm. **(F and G)** (F) Number of Olig2^+^ cells/mm^2^ in brain and (G) SC tissues of indicated groups at day 0 (D0), day 10 (D10), and day (D21) after infection. **(H)** Left: Representative immunostainings for NogoA in brain corpus callosum section at D21 in indicated groups. Arrowheads indicate NogoA^+^ cells. Scale bars, 50 μm. Right: Number of NogoA^+^ cells/mm^2^ in brain corpus callosum section at D21 after infection. **(I)** Left: Representative immunostainings for APP in brain sections at D21 in indicated groups. Arrowheads indicate axonal damage. Scale bars, 100 μm. Right: Number of APP^+^ cells/mm^2^ in total brain at D0, D8, and D21 after infection. **(J)** Left: Representative immunostainings for Iba1 in brain section at D21 in indicated groups. Scale bars, 100 μm. Right: Histological percentage of IBA-1^+^ among total brain tissue at D0, D8, and D21 after infection. **(K)** Representative flow cytometry histograms of MHCII, CD86, TMEM119, and CX3CR1 protein expression in microglia at D21 after infection of indicated groups. **(L)** Geometric Mean Fluorescence Intensity (gMFI) of MHCII, CD86, TMEM119. and CX3CR1 in microglia at D21 after infection of indicated groups. ns, not significant; *P ≤ 0.05; **P ≤ 0.01; ***P ≤ 0.001. Images in panels A, E, and H–J were acquired using a whole slide scanner, which automatically stitched together multiple high-resolution image captures; two-tailed unpaired *t* test for C; two-way ANOVA followed by Sidak’s multiple comparisons test for D, F, and G; two-tailed unpaired *t* test for H; two-way ANOVA followed by Fisher’s Least Significant Difference (LSD) multiple comparisons test for I, J, and L. Data represent the pool of two independent experiments (B–G) or are representative of at least two independent experiments (H–J). Bars and horizontal lines represent mean ± SEM. EAE, experimental autoimmune encephalitis.

**Figure S1.**
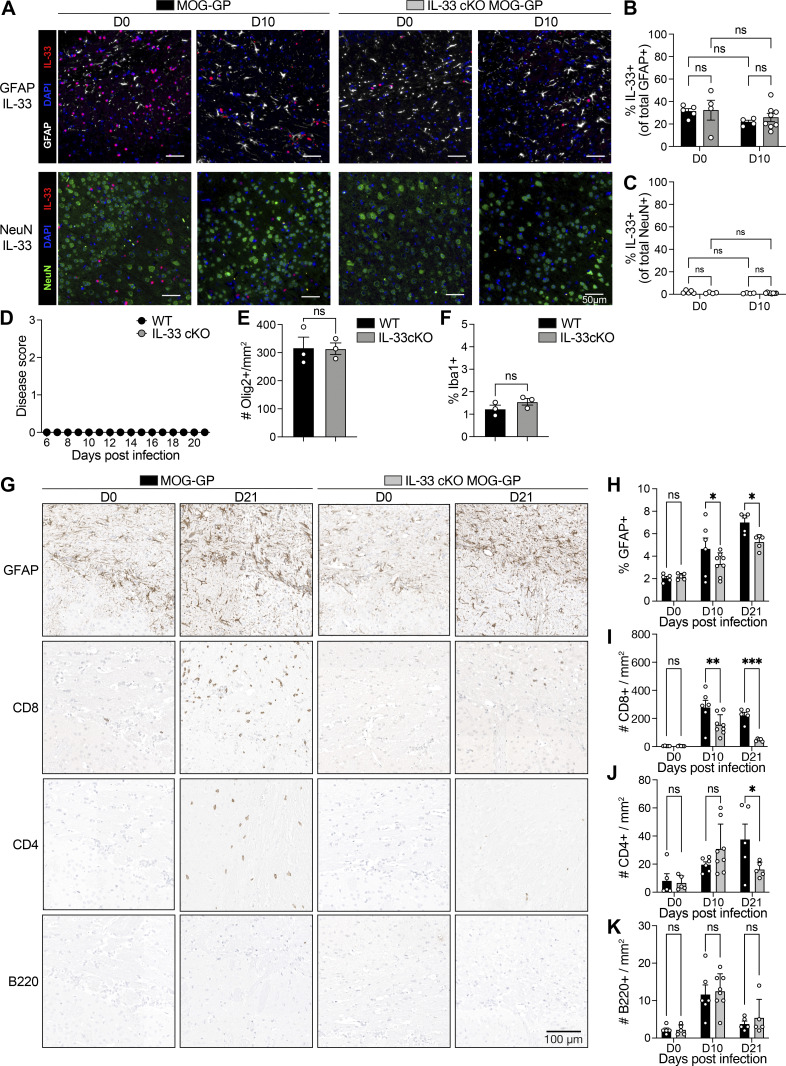
**Absence of oligodendrocyte-derived IL-33 curtails brain inflammation and CD8** ^**+**^ **T cell persistence.** MOG-GP and IL-33 cKO MOG-GP mice were infected i.v. with 10^4^ PFU rLCMV-GP33. **(A)** Brain section of MOG-GP and IL-33 cKO MOG-GP mice stained for (top) GFAP, IL-33, and DAPI or (bottom) NeuN, IL-33, and DAPI at day 0 (D0) and D10 after infection. **(B)** Percentage of IL-33^+^ cells among total GFAP^+^ cells in MOG-GP and IL-33 cKO MOG-GP brain tissue at D0 and D10 after infection. **(C)** Percentage of IL-33^+^ cells among total NeuN^+^ cells in MOG-GP and IL-33 cKO MOG-GP brain tissues at D0 and D10 after infection. **(D)** EAE disease course (*n* = 3 WT mice; *n* = 3 IL-33 cKO mice); clinical scores are expressed as mean ± SEM. **(E)** Quantification of Olig2^+^ cells/mm^2^ in brain tissue of WT and IL-33 cKO mice at D21 after infection. **(F)** Percentage of iba1^+^ cells in brain tissue of WT and IL-33 cKO mice at D21 after infection. **(G)** Representative immunostainings for GFAP, CD8, CD4, and B220 in brain section at indicated time points in indicated groups. Scale bars, 100 μm. **(H)** Frequencies of GFAP^+^ area among total tissue area from brain section at indicated time points in indicated groups. **(I–K)** Quantification of CD8^+^, (J) CD4^+^, and (K) B220^+^ cells per mm^2^ from brain section at indicated time points in indicated groups. ns, not significant; *P ≤ 0.05; **P ≤ 0.01; ***P ≤ 0.001. Images in panels A and G were acquired using a whole slide scanner, which automatically stitched together multiple high-resolution image captures; two-way ANOVA followed by Sidak’s multiple comparisons test for B, C, and H–K; two-tailed unpaired *t* test for E and F. Data represent the pool of two independent experiments (A–C and H–K). Bars and horizontal lines represent mean ± SEM. EAE, experimental autoimmune encephalitis.

We set out to revisit the cellular sources of IL-33 and its expression levels in the CNS of healthy adult mice. Immunofluorescent co-labeling of IL-33 and Olig2—a TF primarily expressed in oligodendrocyte precursor cells (OPCs) and mature oligodendrocytes—revealed a substantial co-localization of these markers (Fig. 1 A). Consistent with earlier findings (Gadani et al., 2015), oligodendrocyte lineage cells (Olig2^+^) were the predominant source of IL-33 accounting on average for 64.17% and 70.49% of IL-33 expressing cells in the brain and spinal cord, respectively (Olig2^+^ of IL-33^+^; Fig. 1 B). In contrast, 31.3% of total astrocytes and only 2.1% of neurons were IL-33^+^ (Fig. S1, A–C). Hence, we generated a conditional knockout mouse strain lacking IL-33 selectively in mature oligodendrocytes (referred to as “IL-33 cKO mice”, Fig. 1 A). We crossbred mice engineered to express Cre recombinase as a knock-in under control of the myelin oligodendrocyte glycoprotein (GP) (MOG) promoter (MOGiCre [Hövelmeyer et al., 2005]) to mice carrying a floxed IL-33 gene (Chen et al., 2015). When compared with their WT counterpart, IL-33 cKO mice exhibited a profound decrease in the proportion of IL-33^+^ cells among Olig2^+^ oligodendrocytes (Fig. 1 C).

Next, we determined the role of oligodendrocyte-derived IL-33 in CD8^+^ T cell–mediated CNS autoimmune disease by crossing IL-33 cKO mice with the MOG-GP line (Page et al., 2019). MOG-GP mice express the lymphocytic choriomeningitis virus (LCMV) GP (LCMV-GP) as a neo–self-antigen specifically in oligodendrocytes. To initiate an LCMV-GP neo-self–specific CD8^+^ T cell response, we infected these mice either IL33 competent (MOG-GP) or deficient (IL-33 cKO MOG-GP) i.v. with an attenuated variant of LCMV, referred to as “recombinant LCMV (rLCMV)-GP33” (Page et al., 2019). MOG-GP mice are not immunologically tolerant to LCMV-GP expressed in their oligodendrocytes, and rLCMV-GP33 infection elicits a polyclonal endogenous CD8^+^ T cell response against LCMV-GP–expressing oligodendrocytes, causing oligodendrocyte loss and demyelination (Page et al., 2019). Following rLCMV-GP33 infection, IL-33 cKO MOG-GP mice exhibited a significantly milder CNS disease course than IL-33–competent MOG-GP controls (Fig. 1 D). WT and IL-33 cKO mice lacking the MOG-GP transgene remained disease free throughout the entire observation period (Fig. 1 D and Fig. S1 D), with no significant differences in Olig2^+^ cell numbers or ionized calcium-binding adapter molecule 1 (Iba-1^+^) microglia/macrophage area (Fig. S1, E and F). In contrast, diseased MOG-GP mice showed significantly lower Olig2^+^ cell numbers in the brain than IL-33 cKO MOG-GP mice at day 10 (Fig. 1, E and F) but not at day 21 in the brain and spinal cord (Fig. 1, E–G). To address if restored Olig2^+^ numbers could have been due to OPCs proliferation in response to mature oligodendrocytes loss (Mason et al., 2004; Wolswijk, 2000), we stained brain sections collected at day 21 for NogoA, a marker for maturing oligodendrocytes rather than OPCs (Pernet et al., 2008). The corpus callosum of IL-33 cKO MOG-GP mice showed significantly more NogoA-positive oligodendrocytes than the one of MOG-GP mice (Fig. 1 H), suggesting that similar Olig2^+^ cell numbers observed at this later time point (see Fig. 1 F) represented proliferated OPCs, that had not yet matured. Of note, the proportions of IL-33^+^ cells among astrocytes and neurons remained in similar range as MOG-GP and IL33-cKO MOG-GP mice progressed to disease (Fig. S1, A–C).

**Figure S2.**
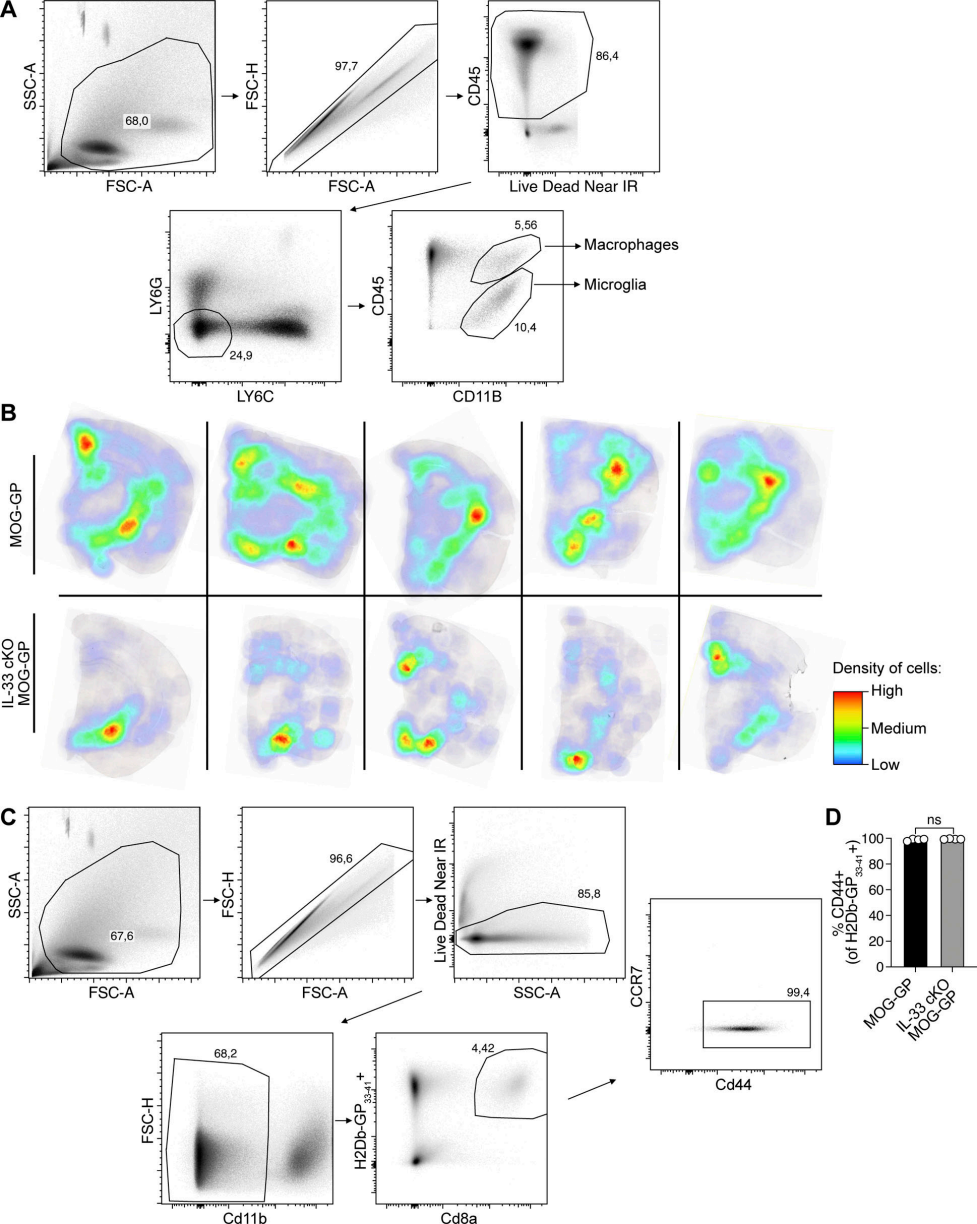
**Gating strategy for macrophages, microglia, and infiltrated CD8** ^**+**^ **T cells in inflamed CNS. (A)** Flow cytometry gating strategy for the identification of macrophages and microglia. Microglia were selected as Ly6G^−^, L6C^−^, CD45^int^, and CD11b^+^ population. Macrophages were selected as Ly6G^−^, L6C^−^, CD45^high^, and CD11b^+^ population. **(B)** Representative density map of brain-infiltrating CD8^+^ T cells in MOG-GP and IL-33 cKO MOG-GP mice at day 21 (D21) after rLCMV-GP33 infection (blue represents low density and red represents high density of infiltrated CD8^+^ T cells). **(C)** Flow cytometry gating strategy for the identification of CD44^+^ H2Db-GP_33–41_specific CD8^+^ T cell population. **(D)** Frequencies of CD44^+^ H2Db-GP_33–41_specific CD8^+^ T cells at D21 after infection. Images from panel B were acquired using a whole slide scanner, which automatically stitched together multiple high-resolution image captures. Symbol represent an individual mouse for C (B and C: *n* = 4 MOG-GP; *n* = 4 IL-33 cKO MOG-GP). ns, not significant. Unpaired two-tailed *t* test for C. Bars and horizontal lines represent mean ± SEM.

Next, we evaluated alterations in the inflamed CNS. Staining against amyloid precursor protein (APP), a marker for axonal injury, revealed fewer injured axonal spheroids in IL-33 cKO MOG-GP mice than in MOG-GP mice at day 21 (Fig. 1 I). We further examined Iba1^+^ microglia/macrophages and glial fibrillary acidic protein (GFAP^+^) astrocytes. Compared with MOG-GP mice, IL-33 cKO MOG-GP mice exhibited a reduced tissue area occupied by Iba1^+^ microglia/macrophages (Fig. 1 J) and GFAP^+^ astrocytes (Fig. S1, G and H), respectively, both at day 10 and day 21. To further assess activation states of microglia, we performed flow cytometry on day 21 in the two cohorts (for gating strategy see Fig. S2 A). Microglia of MOG-GP mice expressed significantly higher levels of activation markers such as MHCII and CD86 than microglia of IL-33 cKO MOG-GP mice. In contrast, homeostatic microglial markers were lower in the latter (Fig. 1, K and L).

Together, these findings suggest that oligodendrocyte-derived IL-33 exacerbates the severity of CNS autoimmune disease and associated tissue immunopathology, as demonstrated by increased axonal damage, increased microglia activation, and more pronounced astrogliosis.

### IL-33 plays a critical role in shaping the transcriptional landscape of autoreactive CD8^+^ T cells during CNS autoimmunity

**Figure 2.**
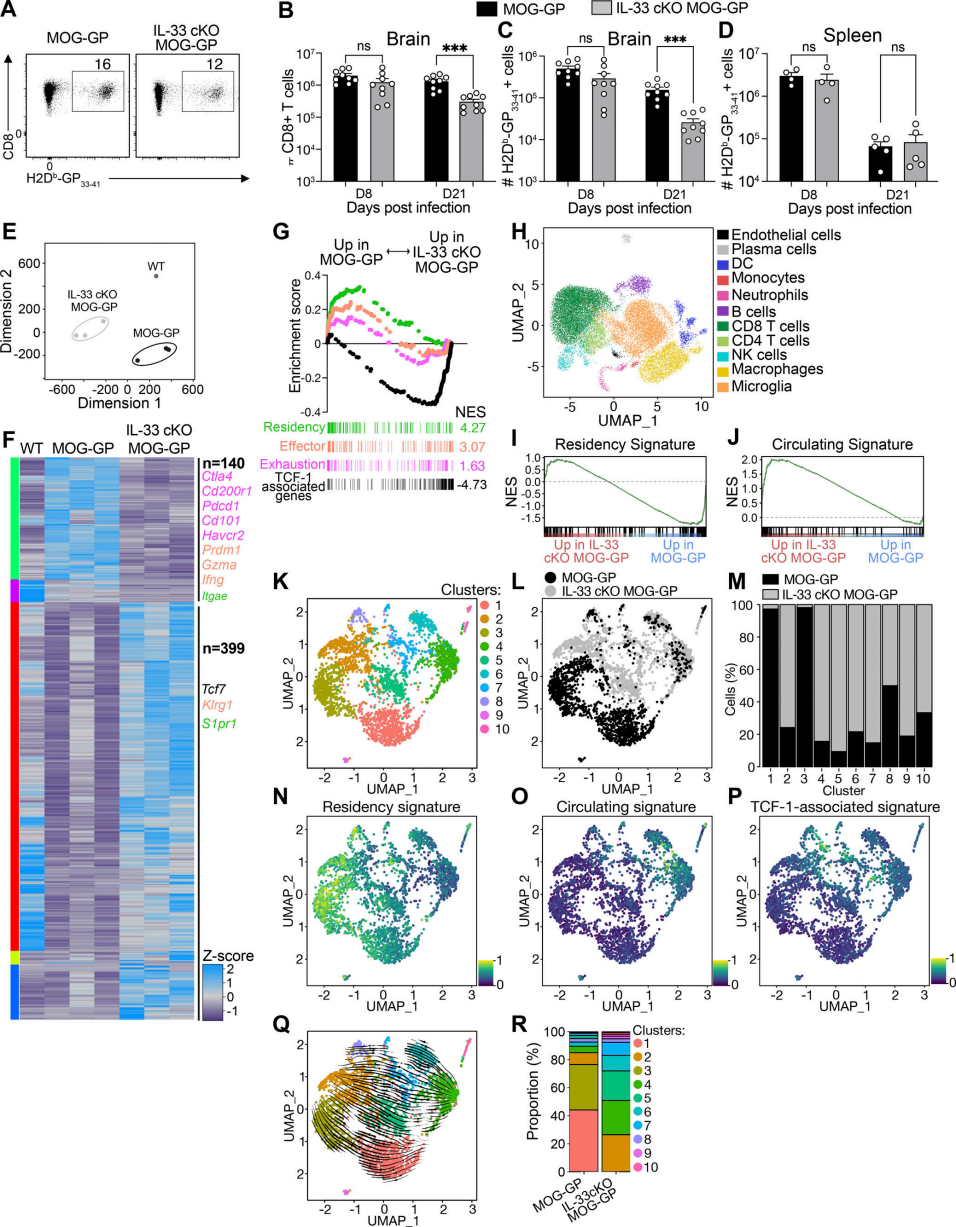
**IL-33 plays a critical role in shaping the transcriptional landscape of autoreactive CD8** ^**+**^ **T cells during CNS autoimmunity. (A–D)** MOG-GP and IL-33 cKO MOG-GP mice were infected i.v. with 10^4^ PFU rLCMV-GP33. **(A)** Representative flow cytometry dot plot of brain-infiltrating H-2D^b^-GP_33–41_–specific CD8^+^ T cells at day 8 (D8) after infection. Numbers over the gate indicate the frequency of H-2D^b^-GP_33–41_–specific CD8^+^ T cells. **(B and C)** (B) Flow cytometric enumeration of brain-infiltrating CD8^+^ T cells and (C) H-2D^b^-GP_33–41_–specific CD8^+^ T cells at D8 and D21 after infection. **(D)** Flow cytometric enumeration of splenic H-2D^b^-GP_33–41_–specific CD8^+^ T cells at D8 and D21 after infection. **(E–G)** RNA-seq of FACS-sorted brain-infiltrating H-2D^b^-GP_33–41_–specific CD8^+^ T cells from WT, MOG-GP, and IL-33 cKO MOG-GP mice at D21 after infection with 10^4^ PFU rLCMV-GP33. **(E)** Multidimensional scaling plot of FACS-sorted H-2Db-GP_33–41_–specific CD8^+^ T cells in indicated groups. **(F)** Heatmap of Z-score of DEGs (FC ≥ 1.5; FDR ≤ 0.05) in MOG-GP versus IL-33 cKO MOG-GP (*n* = 3 mice/group). The corresponding RNA expression of the selected DEGs obtained in WT brain is indicated in the left column of the heatmap (*n* = 1 pool of four mice). **(G)** GSEA of a signature of residency, exhaustion, effector, and TCF-1–associated genes in a ranked list of genes differentially expressed by brain-infiltrating H-2D^b^-GP_33–41_–specific CD8^+^ T cells from MOG-GP versus IL-33 cKO MOG-GP mice. NES: normalized enrichment score. **(H–R)** scRNA-seq on brain-sorted CD45^+^ cells from MOG-GP and IL-33cKO MOG-GP mice at D21 after infection with 10^4^ PFU rLCMV-GP33. **(H)** UMAP of scRNA-seq data. **(I–R)** scRNA-seq analysis on CD8^+^ T cells cluster. **(I and J)** (I) GSEA of a signature of residency and (J) of circulating brain-infiltrated CD8^+^ T cells cluster from MOG-GP versus IL-33 cKO MOG-GP mice. **(K and L)** Seurat clustering of cells identifying (K) 10 clusters or (L) single cells defined by genotype. **(M)** Bar graph showing distribution of cells from indicated groups per cluster identified in K. **(N–P)** UMAP of gene expression of (N) residency, (O) circulating, and (P) TCF-1–associated genes signature on CD8^+^ T cells clusters. **(Q)** UMAP visualization characterized by labeling-based RNA velocity analysis. Arrows represent the integration paths that connect local projections from the observed state to the extrapolated future state. **(R)** Distribution of CD8^+^ T cells among clusters identified in K in MOG-GP and IL-33 cKO MOG-GP mice. ns, not significant; ***P ≤ 0.001 (unpaired *t* test for B–D). Data represent the pool of two independent experiments (B–D) that have been performed once (E–Q). Bars and horizontal lines represent mean ± SEM.

Next, we performed flow cytometry to enumerate GP33-specific T cells in MOG-GP and IL-33 cKO MOG-GP mice at early (day 8) and later (day 21) stages of the disease, both in the CNS and in spleen (Fig. 2, A–D). On day 8, total CD8^+^ T cell and GP_33–41_-specific CD8^+^ T cell counts in the brain were similar in the two experimental groups (Fig. 2, B and C). On day 21, however, IL-33 cKO MOG-GP mice showed significantly lower numbers of GP_33–41_-specific CNS-infiltrating CD8^+^ T cells (Fig. 2, A–C). In contrast, the spleens of the two cohorts harbored similar GP_33–41_-specific CD8^+^ T cell counts at both time point (Fig. 2 D), arguing against an impaired initial expansion of CD8^+^ T cells in secondary lymphoid organs.

Histological enumeration of CNS-infiltrating CD8^+^ T cells revealed a reduced density of CD8^+^ T cells in IL-33 cKO MOG-GP mice from day 10 onward (Fig. S1, G and I). These infiltrates were preferentially situated in white matter regions, where myelinating oligodendrocytes are abundant, and to a lesser extent in the gray matter (Fig. S2 B). Also, CD4^+^ T cell infiltrates were detected in the inflamed CNS, albeit at approximately 10 times lower density than CD8^+^ T cells (Fig. S1, G and J). On day 21, the CD4^+^ T cell density was significantly lower in IL-33 cKO MOG-GP mice than in MOG-GP mice. A few B cells were also observed with no significant difference in abundance between the two genotypes of mice (Fig. S1, G and K).

Next, we explored how oligodendrocyte-derived IL-33 alters the transcriptional landscape of self-reactive CD8^+^ T cells in the inflamed CNS. We performed RNA sequencing (RNA-seq) on FACS-sorted GP_33–41_-specific CD8^+^ T cells from the brains of WT, MOG-GP, and IL-33 cKO MOG-GP mice on day 21 after rLCMV-GP33 infection. Multidimensional scaling analysis of differentially expressed genes (DEGs) segregated GP-reactive CD8^+^ T cells into three distinct clusters corresponding to the three different mouse genotypes (Fig. 2 E). GP_33–41_-specific CNS-infiltrating CD8^+^ T cells from MOG-GP and IL-33 cKO MOG-GP mice showed 539 DEGs (fold change [FC] ≥ 1.5; false discovery rate [FDR] < 0.05; Fig. 2 F). In the absence of oligodendrocyte-derived IL-33, we observed reduced expression of *Itgae* encoding for the retention molecule CD103 and of several inhibitory receptors, including *Pdcd1*, *Havcr2*, *Cd200r1*, *Ctla4*, and *Cd101*. These inhibitory receptors suggest recent antigen contact, and in the context of persistent viral infection, they identify cells in a state termed T cell exhaustion (Fuertes Marraco et al., 2015), but the same receptors are also found in T_RM_ after viral resolution (Wakim et al., 2012). Consistent with these observations, gene set enrichment analysis (GSEA) corroborated that CNS-infiltrating GP_33–41_-specific CD8^+^ T cells from IL-33 cKO MOG-GP mice exhibited lower expression of gene sets associated with tissue residency and effector/exhaustion gene expression (Fig. 2 G). Furthermore, analysis of DEGs revealed for IL-33 cKO MOG-GP mice a relative enrichment of *Tcf7* transcripts encoding for the stemness-related TF TCF-1 (Fig. 2 F). We thus interrogated on a broader scale whether known TCF1-associated genes were enriched in the absence of oligodendrocyte-derived IL-33. GSEA showed that GP_33–41_-specific CD8^+^ T cells in the brain of IL-33 cKO MOG-GP mice exhibited a more pronounced TCF-1–associated gene expression signature as established in chronic viral infection and cancer (Fig. 2 G [Wu et al., 2016]). Flow cytometric analysis confirmed that these T cells isolated from the CNS were not naïve (Fig. S2, C and D), ruling out a potential skewing of bulk RNA-seq results due to high *Tcf-7* expression in naïve T cells (Willinger et al., 2006). Furthermore, we performed single-cell RNA-seq (scRNA-seq) on brain-infiltrating CD45^+^ cells isolated on day 21 from MOG-GP mice with either IL-33–deficient or –sufficient oligodendrocytes (Fig. 2 H). scRNA-seq of the CD8^+^ T cell cluster corroborated that brain-infiltrating CD8^+^ T cells of IL-33 cKO MOG-GP mice exhibited reduced residency but increased circulating gene signatures (Fig. 2, I and J).

**Figure S3.**
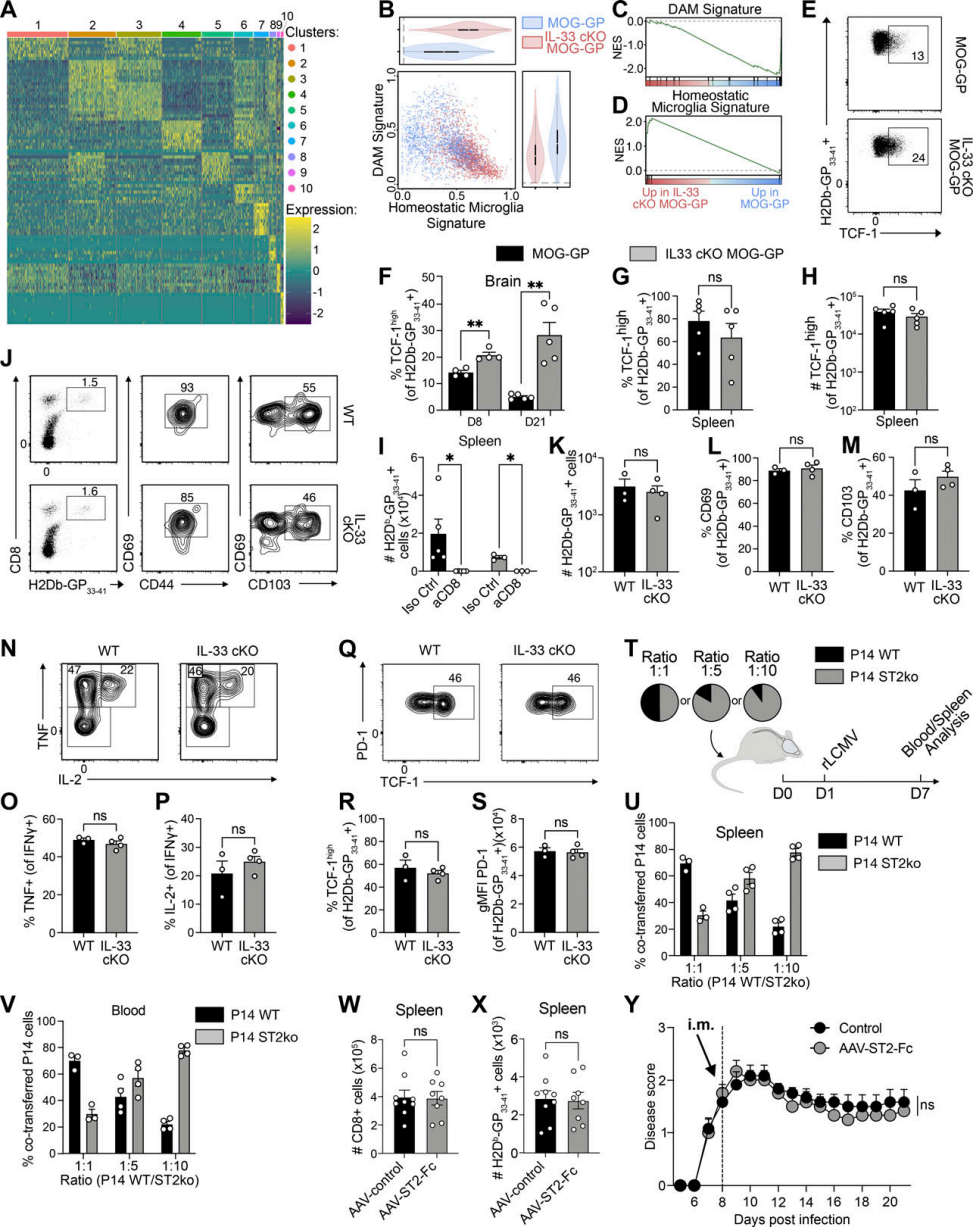
**Characterization of brain inflammation and impact of oligodendrocyte-derived IL-33 on CD8** ^**+**^ **T cell infiltration in MOG-GP and IL-33cKO MOG-GP during resolved transient viral infection and autoimmunity. (A)** Heatmap of top 10 markers of each CD8^+^ T cells cluster in MOG-GP versus IL-33 cKO MOG-GP mice (*n* = 1 mice/group). **(B)** Dot plot of significant enrichment of DAM signature versus homeostatic microglia signature in MOG-GP and IL-33 cKO MOG-GP mice. **(C and D)** GSEA of (C) DAM signature and (D) homeostatic microglia signature in MOG-GP and IL-33 cKO MOG-GP mice. **(E–H)** MOG-GP and IL-33 cKO MOG-GP mice were challenged i.v. with 10^4^ PFU rLCMV-GP33, and brain and splenic H-2D^b^-GP_33–41_–specific CD8^+^ T cells were analyzed at day 8 (D8) and D21 after infection. **(E)** Representative flow cytometry dot plot of TCF-1 expression in brain-infiltrating H-2D^b^-GP_33–41_–specific CD8^+^ T cells at D8 after infection in MOG-GP and IL-33 cKO MOG-GP mice. **(F)** Percentage of TCF-1^high^ cells among brain-infiltrating H-2D^b^-GP_33–41_–specific CD8^+^ T cells at D8 and D21 after infection. **(G and H)** (G) Percentage and (H) absolute count of splenic TCF-1^high^ H-2D^b^-GP_33–41_–specific CD8^+^ T cells at D21 after infection. **(I)** Enumeration of H-2D^b^-GP_33–41_–specific CD8^+^ T cells in the spleen at D28 after infection of indicated groups after a peripheral aCD8 treatment at D21 and D23. Iso Ctrl = isotype control. **(J–S)** WT and IL-33 cKO mice were challenged i.c. with 10^4^ PFU rLCMV-GP33 and brain-derived H-2D^b^-GP_33–41_–specific CD8^+^ T cells were isolated for FACS analysis at 6 wk after infection. **(J)** Representative flow cytometry gating of H-2D^b^-GP_33–41_–specific CD8^+^ T cells and their respective CD69, CD103, and CD44 expression. Numbers indicate the frequency of positive cells. **(K–M)** (K) Absolute counts of H-2D^b^-GP_33–41_–specific CD8^+^ T cells and percentages of (L) CD69^+^ and (M) CD103^+^ cells within the tetramer-specific population. **(N)** Representative flow cytometry plots of intracellular staining for TNF and IL-2 after in vitro stimulation with KAVYNFATC peptide. Cells were gated on IFNγ^+^ H-2D^b^-GP_33–41_–specific CD8^+^ T cells. Numbers indicate the frequency of cytokine-producing cells within each gate. **(O and P)** (O) Frequencies of TNF^+^ cells and (P) IL-2^+^ cells among IFNγ^+^ H-2D^b^-GP_33–41_–specific CD8^+^ T cells. **(Q)** Representative flow cytometry plot of PD-1 and TCF-1 expression in H-2D^b^-GP_33–41_–specific CD8^+^ T cells. **(R)** Frequencies of TCF-1^high^ cells and (S) PD-1^+^ cells among H-2D^b^-GP_33–41_–specific CD8^+^ T cells. **(T)** Experimental design representing the co-transfer of P14 (WT and ST2ko) at different ratios (1:1, 1:5, and 1:10) in MOG-GP recipient mice, followed by rLCMV-GP33 infection at D1 and analysis of blood and spleen at D7. **(U)** Frequencies of co-transferred P14 (WT and ST2ko) at indicated ratio in the spleen. (*n* = 3 for 1:1, *n* = 4 for 1:4 and 1:10). **(V)** Frequencies of co-transferred P14 (WT and ST2ko) at indicated ratio in the blood. (*n* = 3 for 1:1, *n* = 4 for 1:4 and 1:10) **(W–Y)** MOG-GP mice were challenged i.v. with 10^4^ PFU rLCMV-GP33 and treated at D8 after infection with AAV-ST2-Fc or the AAV8-control by i.c. (W and X) or intramuscular (i.m.) (Y). **(W and X)** Flow cytometric enumeration of splenic CD8+ (W) and H-2D^b^-GP_33–41_–specific CD8^+^ T cells (X) at D21 after infection after i.c. treatment. **(Y)** EAE disease course of i.m. after challenge treatment (*n* = 6 MOG-GP mice treated with AAV-ST2-Fc; *n* = 6 MOG-GP mice injected with AAV-control); clinical scores are expressed as mean ± SEM. ns, not significant; *P ≤ 0.05 **; P ≤ 0.01 (score distribution significant with Kolmogorov–Smirnov test for B; two-tailed unpaired *t* test for F–H; two-way ANOVA followed by Fisher’s LSD multiple comparisons test for I; two-tailed unpaired *t* test for K–S; two-tailed unpaired *t* test for W–X; two-way ANOVA followed by Sidak’s multiple comparisons test for Y). Data are representative of at least two independent experiments (E–H and J–S). Bars and horizontal lines represent mean ± SEM. DAM, disease-associated microglia; EAE, experimental autoimmune encephalitis.

Unsupervised clustering of CD8^+^ T cells identified 10 distinct clusters (Fig. 2 K and Fig. S3 A), revealing a transcriptional segregation between IL-33–sufficient and IL-33 cKO MOG-GP mice (Fig. 2 L). Clusters 1 and 3 were dominated by MOG-GP mice, while cells from IL-33 cKO MOG-GP mice dominated clusters 2, 4, 5, 6, 7, 9, and 10 (Fig. 2, L and M). Cluster 8 contained cells from both genotypes (Fig. 2 M). Clusters 1, 2, 3, and 8 predominantly featured the residency signature (Fig. 2 N), while clusters 4, 5, 6, and 7, enriched for circulating and TCF-1–associated gene signatures, were primarily populated by CD8^+^ T cells from IL-33 cKO MOG-GP mice (Fig. 2, O and P).

Consistent with their enhanced residency gene signature, GP_33–41_-specific CD8^+^ T cells of MOG-GP mice exhibited a trajectory evolving from cluster 1 toward cluster 3 (Fig. 2 Q). In contrast, CD8^+^ T cells from IL-33 cKO MOG-GP mice followed a distinct trajectory, initiating from cluster 4 and progressing toward cluster 2 aligning with an altered differentiation program. GP_33–41_-specific CD8^+^ T cells with a residency signature (clusters 1 and 3) constituted 76.6% of the population in MOG-GP mice, whereas the corresponding cluster 2 in IL-33 cKO MOG-GP mice accounted for only 26.5% of brain-infiltrating CD8^+^ T cells (Fig. 2 R).

Furthermore, scRNA-seq analysis restricted to microglia cells showed that in the absence of oligodendrocyte-derived IL-33, the disease-associated microglia (DAM) signature was reduced while the homeostatic microglia transcriptional signature was increased (Fig. S3, B–D), corroborating our observations of decreased microglial activation in the absence of oligodendrocyte-derived IL-33 (see Fig. 1, J–L).

Together, this suggests that oligodendrocyte-derived IL-33 shapes the transcriptional landscape of autoreactive CD8^+^ T cells in the autoimmune CNS by shifting transcriptional effector, exhaustion, circulating and residency signatures, and microglia activation.

### IL-33 promotes the persistence of self-reactive CD8^+^ T cells in the CNS by driving their effector differentiation from TCF-1^high^ stem-like precursors

**Figure 3.**
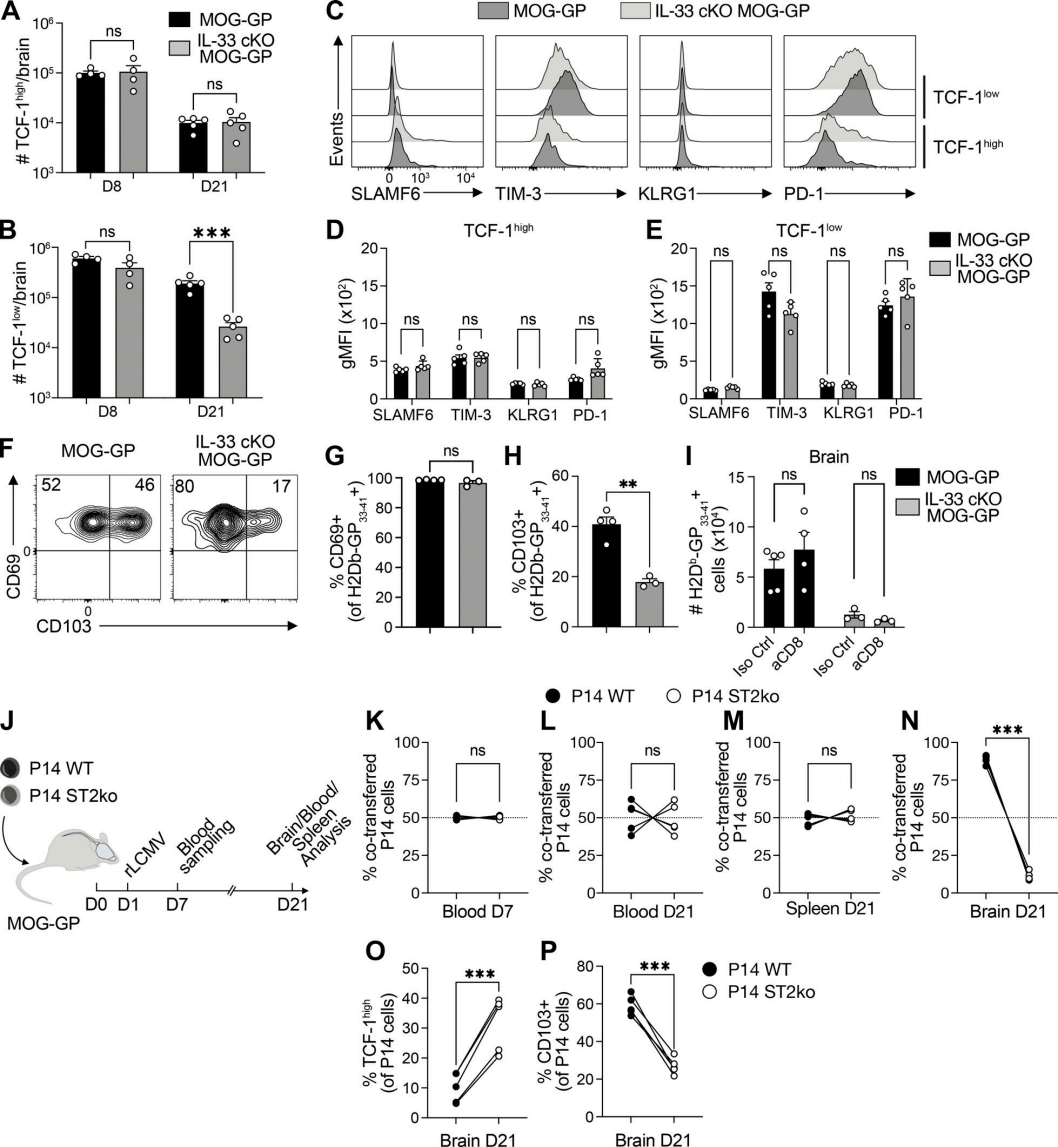
**IL-33 promotes the persistence of self-reactive CD8** ^**+**^ **T cells in the CNS by driving their effector differentiation from TCF-1** ^**high**^ **stem-like precursors. (A–H)** MOG-GP and IL-33 cKO MOG-GP mice were infected i.v. with 10^4^ PFU rLCMV-GP33. **(A and B)** Absolute quantification of (A) TCF-1^high^ and (B) TCF-1^low^ H-2D^b^-GP_33–41_–specific CD8^+^ T cells at day 8 (D8) and D21 after infection. **(C)** Representative flow cytometry histograms of the indicated protein expression in brain-infiltrating TCF-1^high^ and TCF-1^low^ H-2D^b^-GP_33–41_–specific CD8^+^ T cells at D21 after infection. **(D and E)** gMFI of SLAMF6, TIM-3, KLRG1, and PD-1 in TCF-1^high^ (D) and TCF-1^low^ (E) H-2D^b^-GP_33–41_–specific CD8^+^ T cells at D8 and D21 after infection. **(F)** Representative flow cytometry dot plot of CD69 and CD103 expression in brain-infiltrating H-2D^b^-GP_33–41_–specific CD8^+^ T cells at D21 after infection. **(G and H)** Frequencies of (G) CD69^+^ and (H) CD103^+^ H-2D^b^-GP_33–41_–specific CD8^+^ T cells at D21 after infection. **(I)** Enumeration of H-2D^b^-GP_33–41_–specific CD8^+^ T cells in the brain at D28 after infection of indicated groups after a peripheral aCD8 treatment at D21 and D23. Iso Ctrl = isotype control. **(J–P)** MOG-GP mice were co-transferred with P14 (WT and ST2ko) followed by an i.v. infection with 10^4^ PFU rLCMV-GP33 (*n* = 5 recipient mice). **(J)** Experimental procedure: Mice were co-transferred with P14 (WT and ST2ko) on D0 and subsequently infected with rLCMV-GP33 at D1. Blood was collected at D7 and brain, blood, and spleen at D21. **(K)** Frequencies of co-transferred P14 cells (WT and ST2ko) in blood of MOG-GP mice D7 after infection. **(L–N)** Frequencies of co-transferred P14 cells (WT and ST2ko) in (L) blood, (M) spleen, and (N) brain of MOG-GP recipient mice at D21 after infection. **(O)** Frequencies of TCF-1^high^ co-transferred P14 cells (WT and ST2ko) in the brain of MOG-GP recipient mice at D21 after infection. **(P)** Frequencies of CD103^+^ co-transferred P14 cells (WT and ST2ko) in the brain of MOG-GP recipient mice at D21 after infection. ns, not significant; **P ≤ 0.01; ***P ≤ 0.001 (unpaired *t* test for A, B, G, and H; unpaired *t* test with Benjamini, Krieger, and Yekutieli multiple comparisons test for D–E; two-way ANOVA followed by Fisher’s LSD multiple comparisons test for I; two-tailed paired *t* test for K–P). Data are representative of at least two independent experiments (A–H). Bars and horizontal lines represent mean ± SEM.

We next investigated the impact of oligodendrocyte-derived IL-33 on TCF-1 protein expression in GP_33–41_-specific CNS-infiltrating CD8^+^ T cells. At both early (day 8) and late (day 21) stages of CNS autoimmunity, the percentage of TCF-1^high^ cells among brain-infiltrating GP_33–41_-specific CD8^+^ cells was higher in IL-33 cKO MOG-GP mice than in MOG-GP controls (Fig. S3, E and F). However, the respective absolute cell count was similar in the two groups (Fig. 3 A). Conversely, the pool of TCF-1^low^ effector-differentiated GP_33–41_-specific CD8^+^ T cells in the brains of IL-33 cKO MOG-GP mice on day 8 was at a level similar to the one in MOG-GP control mice but contracted to ∼10-fold lower levels by day 21 (Fig. 3 B). The proportional increase in TCF-1^high^ cells among autoreactive CD8^+^ T cells was restricted to the CNS of IL-33 cKO MOG-GP mice, given that the frequencies and absolute numbers of TCF-1^high^ GP_33–41_-specific CD8^+^ T cells in the spleen were similar to those in MOG-GP mice (Fig. S3, G and H).

We next examined if oligodendrocyte-derived IL-33 affects the expression of the inhibitory receptors SLAMF6, PD-1, TIM-3, and KLRG1 by self-reactive GP_33–41_-specific CD8^+^ T cells. When separately analyzing the TCF-1^high^ and TCF-1^low^ subsets of GP_33–41_-specific CD8^+^ T cells in the brain on day 21, we observed that the absence of IL-33 did not impact inhibitory receptor expression in either subset (Fig. 3, C–E). Moreover, we observed that GP_33–41_-specific CD8^+^ T cells persisting in the brain of these animals expressed lower levels of CD103, while CD69 levels were similar to MOG-GP controls (Fig. 3, F–H). To investigate if there is a disequilibrium between CNS-resident CD8^+^ T cells and their circulating counterparts, we administered depleting anti-CD8 antibody intraperitoneally to eliminate the circulating CD8^+^ T cell starting from day 21. Peripheral CD8^+^ T cell depletion (Fig. S3 I) did not affect GP_33–41_-specific CD8^+^ T cell counts in the CNS of IL-33 cKO MOG-GP and MOG-GP mice (Fig. 3 I), whereas total CD8^+^ T cell counts in the brain of IL-33 cKO MOG-GP were substantially lower than in MOG-GP controls.

We also examined the role of oligodendrocyte-derived IL-33 in the persistence of CD8^+^ brain tissue–resident T cells following transient viral CNS infection using an intracranial (i.c.) rLCMV-GP33 model (Pinschewer et al., 2010; Steinbach et al., 2016). IL-33 cKO and WT mice showed similar numbers of GP_33–41_-specific CD8^+^ T cells, with equivalent expression of CD69, CD103, TCF-1, and PD-1, and similar cytokine production (Fig. S3, J–S), indicating that oligodendrocyte-derived IL-33 is not critical for antiviral CD8^+^ T cell generation or persistence following transient viral CNS infection.

To determine if oligodendrocyte-derived IL-33 acts directly on CD8^+^ T cells via the ST2 receptor during autoimmune conditions, we performed a co-transfer experiment using LCMV-GP_33–41_–specific P14 CD8^+^ TCR transgenic cells in MOG-GP mice. These cells were either ST2 competent (P14 WT) or deficient (P14 ST2ko) (Fig. 3 J). The co-transfer was performed at a cellular ratio designed to ensure that upon priming and expansion in the co-transferred P14 populations reached similar population size in the spleen and in the blood on day 7 and day 21 (Fig. 3, K–M and Fig. S3, T–V). In contrast to these peripheral compartments, the CNS contained significantly fewer P14 ST2ko cells than P14 WT cells (Fig. 3 N). P14 ST2ko cells also exhibited an increased fraction of TCF1^high^ cells and a significantly decreased subset of CD103^+^ cells (Fig. 3, O and P).

Together, these findings indicate that oligodendrocyte-derived IL-33 signals directly via the ST2 receptor on CD8^+^ T cells, governing both their local maintenance and/or expansion and their phenotype in the context of CNS autoimmunity.

### Therapeutic blockade of IL-33 signaling in the CNS mitigates the severity of CNS autoimmune disease

**Figure 4.**
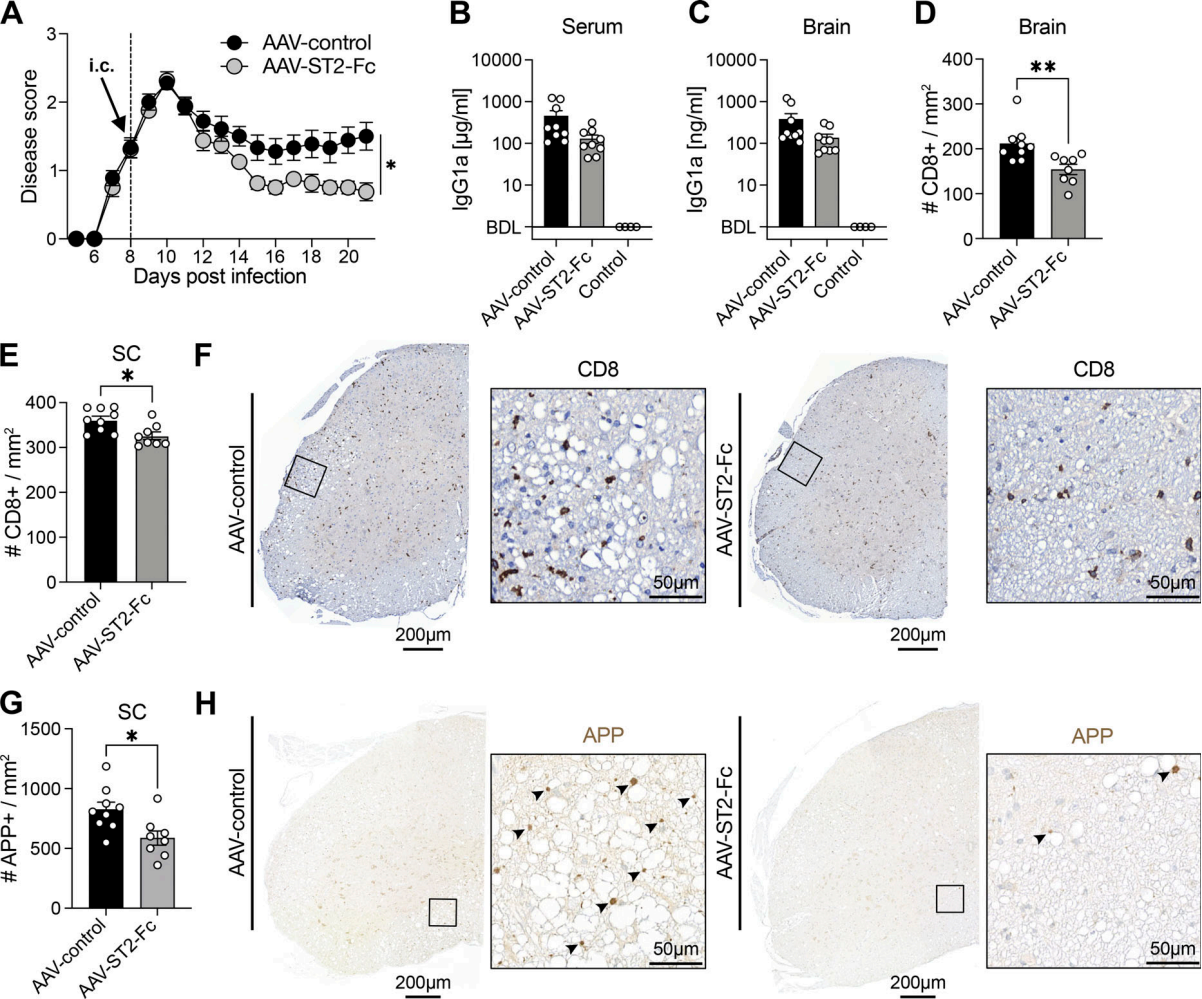
**Local blockade of IL-33 pathway mitigates the severity of CNS autoimmune disease.** MOG-GP mice were challenged i.v. with 10^4^ PFU rLCMV-GP33 and treated 8 days after infection with AAV-ST2-Fc or the AAV8-Vl10 (control) by i.c. (*n* = 9 MOG-GP mice treated with AAV-ST2-Fc; *n* = 8 MOG-GP mice treated with AAV8-Vl10 [control]). **(A)** EAE disease course of i.c. after challenge treatment (*n* = 9 MOG-GP mice treated with AAV-ST2-Fc; *n* = 8 MOG-GP mice treated with AAV8-Vl10 [control]); clinical scores are expressed as mean ± SEM. **(B)** Quantification of ST2-Fc and Vl10 control construct in the serum at day 21 (D21) after infection. **(C)** Quantification of ST2-Fc and Vl10 control construct in the brain at D21 after infection. **(D)** Quantification of CD8^+^ T cell infiltration in brain per mm^2^ after AAV i.c. treatment at D21 after infection. **(E)** Quantification of CD8^+^ T cell infiltration in spinal cord (SC) per mm^2^ after AAV i.c. treatment at D21 after infection. **(F)** Representative immunostainings for CD8^+^ T cell in spinal cord tissue after (left) AAV-control or (right) AAV-ST2-Fc at D21 after infection. **(G)** Quantification of APP per mm^2^ in spinal cord tissue after AAV i.c. treatment at D21 after infection. **(H)** Representative immunostainings for APP in spinal cord tissue after (left) AAV-control or (right) AAV-ST2-Fc at D21 after infection. Arrowheads indicate axonal damage. *P ≤ 0.05; **P ≤ 0.01. Images in panels F and H were acquired using a whole slide scanner, which automatically stitched together multiple high-resolution image captures; two-way ANOVA followed by Sidak’s multiple comparisons test for A; two-tailed unpaired *t* test for B–E and G. Bars and horizontal lines represent mean ± SEM. EAE, experimental autoimmune encephalitis.

We next explored whether IL-33 represents a druggable target in CD8^+^ T cell–driven autoimmune disease of the CNS. We exploited an adeno-associated viral (AAV) vector expressing an IL-33 decoy receptor (AAV-ST2-Fc), which can be administered to mice as somatic gene therapy and blocks IL-33 signaling to CD8^+^ T cells (Kastner et al., 2024). We administered AAV-ST2-Fc i.c. to MOG-GP mice on day 8 after rLCMV-GP33 infection. When compared with controls receiving an AAV control vector expressing irrelevant cargo (AAV-control), AAV-ST2-Fc therapy significantly ameliorated disease progression from around day 14 onward (Fig. 4 A). We confirmed expression of the ST2-Fc decoy receptor in the serum and brain of i.c. injected mice on day 21 (Fig. 4, B and C). CNS-infiltrating CD8^+^ T cells on day 21 were significantly reduced in AAV-ST2-Fc–treated mice (Fig. 4, D–F). Consequently, the number of damaged axons as visualized by staining for APP was significantly lower in the AAV-ST2-Fc–treated group than in AAV-control–treated mice (Fig. 4, G and H). The effect on CD8^+^ T cells was confined to the CNS, as this treatment did not result in a redistribution or in altered T cell counts in the spleen (Fig. S3, W and X). Likewise, intramuscularly administered AAV-ST2-Fc for systemic delivery failed to afford clinical benefit to diseased mice (Fig. S3 Y). Taken together, these data identify IL-33 as a druggable tissue factor in the CNS, opening new avenues to mitigate the long-term progression and outcome of T cell–driven CNS autoimmune disease.

Our study shows that oligodendrocyte-derived IL-33 is important for shaping the differentiation of self-reactive brain-infiltrating CD8^+^ T cells, affecting the disease progression and tissue destruction. The absence of IL-33 from oligodendrocytes resulted in reduced persistence of self-reactive CD8^+^ T cells in the CNS and attenuated transcriptional signatures of tissue residency and effector cell differentiation. Furthermore, in the absence of IL-33 in oligodendrocytes, TCF-1^high^ stem-like CD8^+^ T cells exhibited an impaired capacity to replenish the effector CD8^+^ T cell pool, which is required for sustaining long-term autoimmune disease activity. These results highlight the importance of oligodendrocyte-derived IL-33 during autoimmunity and its potential as a therapeutic target in autoimmune diseases.

We previously demonstrated that self-reactive CD8^+^ T cells in the CNS undergo a distinct differentiation trajectory, characterized by the acquisition of an exhausted-like phenotype (Page et al., 2021). Consistent with a recent study (Grebinoski et al., 2022), our findings suggest that IL-33 regulates the stemness activity of autoreactive CD8^+^ T cells by which the pool of effector self-reactive CD8^+^ T cells is replenished and thus relies on stem-like progenitors in the affected tissue. This contrasts with findings of a mouse diabetes model, where pathogenic effector CD8^+^ T cell population originates primarily from a CD8^+^ T cell population located in the peripheral lymph node at distance from the antigenic source (Abdelsamed et al., 2020; Gearty et al., 2022). This discrepancy may stem from differences in tissue microenvironments in which compartmentalized immune responses in the CNS may prevail as the disease progresses toward chronicity (Matejuk et al., 2021; Forrester et al., 2018). In line with previous observations (Casey et al., 2012), our study emphasizes the critical role of IL-33 in promoting the differentiation of T cells into tissue-resident T cells. IL-33 can act on the ST2 receptor on microglia in the CNS (Wheeler et al., 2023), raising the possibility that IL-33 could affects CD8^+^ T cell differentiation indirectly by IL-33 signaling on microglia and impacting the overall inflammatory milieu. Our data suggest that an IL-33 from oligodendrocyte directly signals to ST2 receptor–expressing CD8^+^ T cells, supported by our observation that ST2 receptor–deficient autoreactive CD8^+^ T cells recapitulated similar differentiation and residency patterns as in MOG-GP IL-33 cKO mice. Furthermore, IL-33 signaling may enhance differentiation, cytokine expression, and expansion of self-reactive CD8^+^ T cells in the CNS, potentially leading to microglial activation and further tissue damage.

TCF-1 inhibits the tissue residency program of memory CD8^+^ T cells in the lung, mainly by repressing the expression of the retention molecule CD103 (Wu et al., 2020). Our observations suggest that in the absence of IL-33 in oligodendrocytes, autoreactive CD8^+^ T cells remain preferentially trapped in a TCF-1^high^ stem-like cell stage, resulting in a compromised tissue-resident core signature marked by reduced CD103 expression. This appears to be particularly relevant in the context of chronic autoimmune conditions, where autoreactive T cells likely encounter antigens over a prolonged period. In addition to the role of clonal expansion of self-reactive CD8^+^ T cells targeting antigenic oligodendrocytes, oligodendrocyte-derived IL-33 may impact the emergence of new T cell clones through antigen release and epitope spreading, which may perpetuate inflammation and disease progression in MS.

IL-33 initiates signaling cascades in context of T cell activation, differentiation, and expansion in secondary lymphoid tissues (Guo et al., 2022; Brunner et al., 2024). The finding that oligodendrocyte-derived IL-33 influences T cell fate bifurcation at the inflamed tissue underscores its potential for future therapeutic interventions. Targeting IL-33 signaling with an AAV-delivered decoy receptor could diminish compartmentalized inflammation in more advanced disease stages in MS (Allan et al., 2016) and could specifically address the pathogenic contribution by tissue-resident CD8^+^ T cells (Vincenti et al., 2022). A previous study showed that administration of a blocking anti-IL-33 antibody during the induction phase of experimental autoimmune encephalitis inhibited the onset and severity of the disease course (Li et al., 2012). Given the established role of IL-33 in the context of T cell priming inside secondary organs (Marx et al., 2023), preventive treatment approaches may imperfectly recapitulate the therapeutic setting of IL-33 modulation in human CNS autoimmune diseases, where intervention occurs after the initial T cell priming. We observed a therapeutic benefit, when administering an AAV-based IL-33 decoy receptor i.c. but not intramuscularly. Further, the i.c. treatment specifically reduced T cell numbers within the CNS without affecting those in the peripheral compartment. These findings collectively suggest that the IL-33 decoy receptor treatment exerted its beneficial effects within the tissue compartments of the CNS.

In summary, our study provides new insights into the role of oligodendrocyte-derived IL-33, which facilitates the differentiation of self-reactive TCF-1^high^ stem-like cells into terminally differentiated effector cells of high encephalitogenic potential and may thus contribute to defining novel immunotherapeutic strategies for chronic CNS autoimmune disorders.

## Materials and methods

### Mice

C57BL/6J WT was obtained from Charles River. The C57BL/6J MOG-GP (MOGi^Cre/+^: Stop-GP^flox/+^) mouse line was generated by crossing mice expressing the Cre-recombinase under the control of the oligodendrocyte-specific promoter (C57BL/6J MOGiCre [Hövelmeyer et al., 2005]) with C57BL/6J Stop-GP mice [Page et al., 2019]). The C57BL/6J IL-33 cKO MOG-GP mouse line was generated by crossing IL-33^flox/flox^ mice (Chen et al., 2015) with C57BL/6J MOG-GP mice. All mice were lodged under specific pathogen–free P2 conditions in the animal facilities of the University Medical Center of Geneva. Male and female sex- and age-matched mice between 6 and 12 wk of age were used for experiments. All animal experiments were authorized by the cantonal veterinary office of Geneva and performed in agreement with the Swiss law for animal protection.

### Virus infection

Recombinant LCMV was generated and propagated on baby hamster kidney 21 cells BHK21 (CCL-10; ATCC) (Flatz et al., 2006). Virus stocks were titrated using MC57G cells (CRL-2295-ATCC) according to established methods (Kallert et al., 2017). The recombinant rLCMV-GP33 expresses the signal sequence of LCMV-GP harboring the GP_33–41_ epitope fused to the VSV GP instead of the LCMV full-length GP. For transient virus infection in the brain, 10^4^ PFU of rLCMV-GP33 were diluted in 30 μl of minimum essential medium (Gibco) before i.c. injection. For the peripheral challenge, mice were i.v. injected into the tail vein with 10^4^ PFU of rLCMV-GP33 in 100 μl. Infected animals were monitored daily for the occurrence of classical experimental autoimmune encephalomyelitis symptoms and scored as follows: (1) flaccid tail; (2) impaired righting reflex and hind limb weakness; (3) complete hind limb paralysis; (4) complete hind limb paralysis with partial forelimb paralysis; and (5) moribund. Severely diseased animals were immediately sacrificed.

To block IL-33 signaling, we used an AAV-ST2-Fc described by Kastner et al. (2024). In short, the ST2-Fc construct is composed of a signal peptide and the extracellular domain of ST2 (NCBI, GenBank: M24843.1) coupled C-terminally to the mouse IgG1a constant domain. The ST2-Fc was cloned into the pENN.AAV.CB7.CI AAV expression plasmid, which contains a CAG-promoter–driven expression cassette flanked by AAV2-inverted terminal repeats, obtained from the PENN Vector Core (Perelman School of Medicine, University of Pennsylvania, Philadelphia, PA, USA) under material transfer agreement (MTA). As a control, we used an AAV vector expressing the antibody VI10 (Kalinke et al., 1996; Ertuna et al., 2021). AAV vector production (in an AAV8 capsid format) and titration were performed by the Viral Vector Facility of the Neuroscience Center Zurich, Switzerland. AAV vectors were administered to mice i.c. and i.m. at a dose of 1 × 10^11^ viral genomes per mouse.

### Antibody-mediated depletion

For depletion of circulating CD8^+^ T cells, mice were treated intraperitoneally twice over a 3-day interval with 200 μg of rat-anti-mouse-CD8α–depleting antibody (BE0117; BioXCell) or isotype control (BE0090; BioXCell). Anti-CD8 was administered 21 days after rLCMV infection. Depletion of T cells was confirmed by flow cytometry.

### Immunohistochemistry

Brain and spinal cord tissues were collected and fixed in 4% paraformaldehyde overnight at 4°C. Dehydrated tissues were embedded in paraffin. Deparaffinized tissue sections (2 μm) were incubated with Dako REAL peroxidase-blocking solution (K0672; Dako) to inactivate endogenous peroxidases, and unspecific binding was blocked (PBS/1% BSA). After antigen retrieval, sections were incubated with primary antibody for IL-33 (Cat# AF3626; R&D Systems) and Olig2 (Cat# 18953; R&D Systems). Bound primary antibodies were visualized with anti-goat Alexa Fluor 488–labeled secondary antibodies or peroxidase-labeled secondary antibodies, followed by the Opal 570 signal amplification system (Opal 570; FP1488001KT; Akoya). Nuclei were stained with DAPI (D1306; Invitrogen).

For bright-field microscopic images, bound primary antibodies anti-CD8 (14-0195-80; eBioscience), anti-CD4 (#25229; Cell signaling), anti-B220 (14-0452-85; eBioscience), anti-GFAP (Z0334; Dako), anti-IBA-1(019-1974; Wako), anti-APP (MAB348; Millipore) were visualized using peroxidase-coupled secondary antibody systems (Dako; Vector Laboratories) and polymerized 3,3′-diaminobenzidine (Dako). Nuclei were counterstained with hemalum.

### Image analysis

Immunostained sections were scanned using Pannoramic Digital Slide Scanner 250 FLASH II (3DHISTECH) at 200× magnification. Automatic quantification of positive cells was performed using the Visiopharm platform using a custom-made script. For representative images, the white balance was adjusted, and contrast was enhanced using the tools “levels”, “curves”, “brightness”, and “contrast” in Photoshop CS6 (Adobe). All modifications were acquired uniformly on the entire image.

### Density map

The density map was generated using the software QuPath following cell detection analysis. A consistent threshold was applied across all processed images.

### FACS analysis and sorting

The following fluorophore-conjugated antibodies for flow cytometry were purchased from BioLegend (anti-CD8a Pacific Blue [53-6.7], anti-CD8a Brillant Violet 605 [53-6.7], anti–TIM-3 Brillant Violet 605 [RMT3–23], anti-KLRG1 FITC [2F1], anti–PD-1 PerCP/Cyanine5.5 [29F.1A12], anti-CD69 FITC [H1.2F3], anti-CD103 Alexa Fluor 647 [2E7], anti-CD44 PerCP/Cyanine5.5 [IM7], anti-IFNg Brillant Violet 421 [XMG1.2], anti-IL-2 PE [JES6-5H4], and anti-TNF PE/Cyanine7 [MP6-XT22]), Cell signaling Technology (anti–TCF-1 Alexa Fluor 647 [C63D9]), and BD Biosciences (anti-SLAMF6 Brillant Ultraviolet 395 [13G3]). For detection of GP-specific CD8^+^ T cells, PE-labeled Db-GP_33–41_ tetramer (provided by the National Institutes of Health Tetramer Core Facility) was used. Dead cells were excluded by staining with Zombie NIR dye (BioLegend). Most antibodies were used at a 1/100 dilution. Peripheral blood samples were obtained by facial vein puncture in heparin. Blood erythrocytes were lysed, and cells fixed using BD FACS Lysing Solution (349202; BD Biosciences). For the preparation of CNS-infiltrating leukocytes, mice were anesthetized and transcardially perfused with PBS. Brains were minced and digested in DMEM with collagenase A (1 mg/ml; Roche) and DNase I (0.1 mg/ml; Roche) for 1 h at 37°C and homogenized using 70-μm cell strainers (BD). Leukocytes were separated using a discontinuous percoll gradient (30/70%). The remaining erythrocytes were lysed using RBC Lysis buffer (420301; BioLegend) for 3 min at room temperature. Surface staining was carried out with directly labeled antibodies in FACS buffer (2.5% FCS, 10 mM EDTA, and 0.01% NaN3 in PBS). Isolated cells were quantified using AccuCheck Counting Beads (PCB100; Invitrogen). Intracellular staining of TCF-1 was performed using the FoxP3/Transcription Factor Staining Buffer Set (00-5523-00; eBioscience) according to the manufacturer’s instructions. To assess intracellular cytokine production, isolated leukocytes were cultured for 5 h in the presence of monensin (BioLegend) and brefeldin (BioLegend). Cells were stimulated with 1 μM KAVYNFATM peptide. Flow cytometric samples were acquired on a BD LSRFortessa (BD Biosciences) using BD FACSDiva (BD Biosciences, v8.0.2) and an Attune NxT Acoustic Focusing Cytometer (Thermo Fisher Scientific, V3.1.2) using appropriate filter sets and compensation controls. Gates were assigned according to appropriate control populations. Data were analyzed using FlowJo software (V10).

### RNA-seq sample preparation

To perform RNA-seq, we FACS-sorted brain CD8 GP_33–41_^+^ T cells from WT, MOG-GP, and IL-33 cKO MOG-GP mice at 21 days after rLCMV-GP33 infection. Each biological replicate (*n* = 3 for MOG-GP, *n* = 3 for IL-33 cKO MOG-GP, and *n* = 1 pool of 4 WT mice) represents 10^4^ sorted H-2D^b^-GP_33–41_–specific CD8^+^ T cells in RLT buffer with 1% β-mercaptoethanol and processed with RNAeasy Micro Kit (74004; Qiagen) following the manufacturer’s instructions.

CD8 GP_33–41_^+^ T cells were sorted in RLT buffer with 1% β-mercaptoethanol and processed with RNAeasy Micro Kit (74004; Qiagen) following the manufacturer’s instructions.

Quality and integrity of RNA were assessed with the fragment analyzer by using the standard sensitivity RNA Analysis Kit (Advanced Analytical, DNF-471). All samples selected for sequencing exhibited an RNA integrity number over 8. RNA-seq libraries were generated using 100 ng total RNA of a non-stranded RNA-seq, massively parallel mRNA sequencing approach from Illumina (TruSeq mRNA Library Preparation, 20020594; Illumina). Library preparation was performed on the Beckman Coulter’s Biomek FXP workstation. For accurate quantitation of cDNA libraries, the fluorometric-based system QuantiFluor dsDNA System was used (E2670; Promega). The size of the final cDNA libraries was determined by using the dsDNA 905 Reagent Kit (DNF-905; Advanced Bioanalytical), exhibiting a sizing of 300 bp on average. Libraries were pooled and sequenced on the Illumina HiSeq 4000 sequencer (SE; 1 × 50 bp; 30–35 Mio reads/sample). Basecalls generated by Illumina’s Real Time Analysis software were demultiplexed to FASTQ files with bcl2fastq (v2.17.1.14). The quality check was done using FastQC (Andrews, 2010). https://www.bioinformatics.babraham.ac.uk/projects/fastqc

### Single RNA-seq sample preparation

For scRNA-seq, brain-derived CD45^+^ cells were FACS-sorted from MOG-GP and MOG-GP IL-33 cKO mice 21 days after rLCMV-GP33 infection. Cells were adjusted to 5.5 × 10^5^ cells/ml in PBS, 0.04% BSA, before proceeding with droplet generation.

Single cell 10X libraries were constructed from isolated single cells following the Chromium Single Cell 3′ Reagent Kits (Chromium GEM-X Single Cell 3′ Kit v4). Briefly, single cells were co-encapsulated with gel beads (10X Genomics) in droplets using Chromium Chip G (10X Genomics). Library construction was carried out using the Chromium Single Cell 3’ Library Kit (v4, 10X Genomics). Reverse transcription, preparation of single-cell transcriptome and libraries construction were performed following the manufacturer’s instructions.

Final libraries were pooled and sequenced on Illumina NextSeq 500 platform (PE, 100 cycles) with a concentration of 1.8 pM with 5% PhiX. Resulting FASTQ files were demultiplexed and subsequently used as input to cellranger (10x Genomics).

### RNA-seq data processing

FASTQ reads were mapped to the ENSEMBL reference genome (GRCm38.96) using STAR version 2.4.0j (Dobin et al., 2013) with standard settings except that any reads mapping to more than one location in the genome (ambiguous reads) were discarded (outFilterMultimapNmay = 1).

A unique gene model was used to quantify the number of reads per gene. Briefly, the model considers all annotated exons of all annotated protein-coding isoforms of a gene to create a unique gene where the genomic regions of all exons are considered to come from the same RNA molecule and merged together.

All reads overlapping the exons of each unique gene model were reported using featureCounts version 1.4.6-p1 (Quinlan and Hall, 2010). Gene expressions were reported as raw counts and in parallel normalized in reads per kilobase per million mapped reads (RPKM) to filter out genes with low expression values (1 RPKM) before calling for DEGs. Library size normalizations and differential gene expression calculations were performed using the package edgeR (v3.28.0) (Robinson et al., 2010) designed for the R software (v3.6.2). Only genes having a significant FC (Benjamini–Hochberg corrected P value <0.05) were considered for the rest of the RNA-seq analysis.

### Single RNA-seq data processing

To get counts, R1 reads were used to identify both cell barcodes and unique molecular identifiers (UMI) using UMI-tools version 1.0.1 (Smith et al., 2017) with the following settings: “--bc-pattern = ’(?P<cell_1>.{16})(?P<umi_1>.{12})’ --extract-method = regex --knee-method = distance --method = umis --set-cell-number = 15000 –ed-above-threshold = discard.”

After read extraction and addition of cell barcodes and UMI to headers of R2 reads, FASTQ reads were mapped to the ENSEMBL reference genome (GRCm38.96) (http://www.ensembl.org/Mus_musculus/Info/Index) using STAR version 2.7.1a (Dobin et al., 2013) with standard settings except that any reads mapping to more than one location in the genome (ambiguous reads) were discarded (m = 1). All reads mapping in the proper orientation within exons and introns were reported using featureCounts version 1.6.4 (Liao et al., 2014). UMI counts were calculated using UMI-tools with “--per gene --gene-tag = XT --assigned-status-tag = XS --per-cell” arguments.

For cell sorting, count matrices were imported and analyzed using Seurat v5 (Hao et al., 2024) package in R version 4.2.3 (https://www.r-project.org/). Only cells with >1,000 UMIs and <0.01% of mitochondrial content were kept for the analysis. Genes quantified in only one cell were removed. The distribution of detected genes per cell was used to manually set threshold to remove putative doublets as well as dead cells. Selection of cells was done with the subset function. As a result, 21,415 cells were kept for the analysis.

For cell type prediction, a sketch-based subsampling of 5,000 cells per condition was used for cell type predictions, following the ‘sketch-based analysis in Seurat v5’ vignette (Sun et al., 2019). 14 atlases of neuronal and non-neuronal cells were used: scMCA (https://satijalab.org/seurat/articles/seurat5_sketch_analysis), all datasets present in the celldex package for R (https://github.com/LTLA/celldex/), as well as two versions—trimmed means and medians gene expression grouped per cluster—of mouse (WCandH) and human (M1 and HMCA) atlases from Allen Brain Atlas (https://portal.brain-map.org/), leading to a total atlas of 1,746 annotated cell types split in 6,208 references. Gene IDs not corresponding to the sample’s organism were translated to organism of interest homologous gene IDs using biomaRt (https://bioconductor.org/packages/release/bioc/html/biomaRt.html) package for R. Pearson’s correlations between each sketch cell and cell type present in the atlas were calculated. For cell type with several entries in the whole atlas, best correlation was chosen. Cell types representing <0.05% of the sketch cells were not considered for the rest of the analysis. Cells predicted to be one of the removed cell types were labeled as “ambiguous”. Top 30 DEGs calculated from each pairwise comparison of all retained cell types were obtained with scConsensus package for R (https://github.com/prabhakarlab/scConsensus), and the pool of all these top 30 DEGs was used to generate a shared nearest neighbor graph with the FindNeighbors and FindClusteringTree functions. Walk on dendrogram was performed by iteration of 1 starting from 1 cluster. Clusters were obtained with the cutree function of the dendextend package (https://cran.r-project.org/web/packages/dendextend/vignettes/dendextend.html) designed for R. At each iteration, a trimmed mean (25%) of the Pearson’s correlations of the cells within each cluster was calculated for each retained cell types, and for each cluster, the cell type corresponding to the maximal trimmed mean was assigned to all cells within the cluster to use neighborhood in cell type predictions. In parallel a consensus score was calculated. When all cell identities were identical to the previous iteration, 1 was added to the consensus score (which was originally set to 0 at first). Otherwise, the consensus score was reset to 0. Iterations stopped, and the final cell identities were assigned as soon as the consensus score reached 10. Cell predictions were then projected to the whole dataset with the function ProjectData.

Microglia and T cells sub-analyses were performed using Seurat v5 (Sun et al., 2019). To prevent overrepresentation of a cell type in one condition, same number of T cells (1608) and microglia (1786) were randomly selected for both conditions. DEGs between MOGGP and IL33KO cells were obtained by FindMarkers function with no logFC threshold to report log_2_ fold changes and P values for all genes expressed in at least 0.05% of cells of a population (min.pct = 0.05). Genes were ranked in descending order according to the calculated log_2_ FC, i.e., from the most upregulated to the most downregulated, as input for GSEA (https://www.pnas.org/content/102/43/15545.full.pdf.html). Tested gene sets are in Table S1.

For each tested signature, core enrichment genes were extracted, and scoring per cell was performed as follows: (1) all gene expressions were normalized by their maximal value, (2) all core gene set mean expressions were calculated for each cell, and (3) finally, for each gene set, values were rescaled from 0 to 1 so all gene set scores have the same range within the whole population. Score distributions were compared using Kolmogorov–Smirnov.

T cells data were used for unsupervised clustering using a cluster resolution of 0.4. Uniform manifold approximation projection (UMAP) was calculated using the first 30 dimensions. Differential gene expression analysis between clusters was performed using the Wilcoxon rank-sum test in the FindMarkers function. Top 10 markers of each cluster were used to generate an heatmap.

Velocity was inferred using Velocyto (La Manno et al., 2018).

### GSEA

GSEA was performed with gene sets identified using published microarray data sets. Effector and exhaustion signatures originated from (Doering et al., 2012) studying the transcriptome of distinct CD8^+^ T cell differentiation states after LCMV Armstrong and LCMV clone 13 infection. TCF-1–enriched gene signatures originated from microarray analysis of TCF-1–overexpressing CD8^+^ T cells (Wu et al., 2016). Residency signature originated from studying the role of RUNX3 on CD8^+^ T cell residency in nonlymphoid tissues and tumors (Milner et al., 2017).

### Statistical analysis

Statistical parameters, including the exact value of *n*, the dispersion, the precision of measures, and the statistical significance, are reported in the figures and figure legends. In figures, asterisks denote statistical significance as calculated by Student’s *t* test and two-way ANOVA with Šidák post-test (ns, not significant; *, P < 0.05; **, P < 0.01; ***, P < 0.001). Statistical analysis was performed in GraphPad Prism 10 and R.

### Online supplemental material

Fig. S1 demonstrates that the absence of oligodendrocyte-derived IL-33 attenuates brain inflammation and reduces the persistence of CD8^+^ T cells. Fig. S2 presents the gating strategy used to identify macrophages/microglia and infiltrated CD8^+^ T cells in an inflamed brain. Additionally, it includes a density map of brain-infiltrating CD8^+^ T cells, which are identified as non-naïve CD8^+^ T cell. Fig. S3 provides the characterization of brain inflammation in MOG-GP and IL-33 cKO MOG-GP models. It also illustrates the impact of oligodendrocyte-derived IL-33 on brain-infiltrating CD8^+^ T cells in resolved transient infection and autoimmunity. Table S1 lists the tested gene sets for the different analyzed signatures.

## Supplementary Material

Table S1 lists the tested gene sets for the different analyzed signatures.

## Data Availability

Data are available in the article and supplementary data file or from the corresponding author upon reasonable request. The raw and processed RNA-seq and scRNA-seq data have been deposited to the Gene Expression Omnibus under accession number: GSE271925.
